# A systems approach to multilingual language attitudes: A case study of Montréal, Québec, Canada

**DOI:** 10.1177/13670069221133305

**Published:** 2023-04-25

**Authors:** Ruo Ying Feng, Mehrgol Tiv, Ethan Kutlu, Jason W. Gullifer, Pauline Palma, Elisabeth O’Regan, Naomi Vingron, Marina M. Doucerain, Debra Titone

**Affiliations:** McGill University, Canada; University of Ottawa, Canada; McGill University, Canada; U.S. Census Bureau, USA; University of Iowa, USA; McGill University, Canada; Lund University, Sweden; McGill University, Canada; University of Québec in Montréal, Canada; McGill University, Canada

**Keywords:** Bilingualism, language attitudes, social network analysis, demographic analysis, social-ecological model, social systems

## Abstract

**Purpose:**

People are shaped holistically by dynamic and interrelated individual and social-ecological systems. This perspective has been discussed in the context of varied aspects of bilingual experiences, namely language acquisition and development. Here, we applied a *Systems Framework of Bilingualism* to language attitudes, which may be especially responsive to social-ecological influences.

**Methodology:**

One hundred twenty-three French–English bilingual adults (*M*_age_ = 21.20, *SD* = 3.21) completed self-report questionnaires on demographic information and their attitudes toward languages. A subset of these bilinguals (*n* = 73) completed a social network survey.

**Data and analysis:**

We used language-tagged social network analysis and geospatial demographic analysis to examine the role of individual characteristics (i.e., first language), *interpersonal language dynamics* (i.e., person-to-person interactions), and *ecological language dynamics* (i.e., neighborhood language exposure).

**Findings and Conclusions:**

At an individual level, we found that bilinguals’ language background (i.e., first language) predicted attitudes of solidarity toward a language (i.e., whether a language is associated with personal identity and belongingness). When considering sociolinguistic layers of influence, we found that bilinguals’ social network and neighborhood-level language exposure jointly predicted their attitudes of solidarity toward a language, as well as their attitudes toward the protection of minority languages.

**Originality:**

While most studies have examined language experience in a unidimensional nature, the present study investigated multilingual language attitudes by considering multiple systems within a social-ecological framework.

**Implications:**

Taken together, the results suggest that several interrelated interpersonal and ecological systems are associated with language attitudes, which could have important implications for planning future language policies in multilingual societies such as Montréal.

People are nested within complex social systems that interact and influence one another (e.g., close relationships, neighborhood, society). This perspective, broadly referred to as a social-ecological or systems framework, has been widely explored in the context of human development (e.g., Bronfenbrenner, 1977). Inspired by this and other related approaches within the language sciences (de Bot, 2008; de Bot et al., 2007; Douglas Fir Group, 2016; Haugen, 1972; Sachdev & Bourhis, 2001; Steffensen & Fill, 2014; Yim et al., 2019), we developed a *Systems Framework of Bilingualism* (Titone & Tiv, 2023a; 2023b; Tiv, Kutlu, Gullifer, et al., 2022) to operationalize the many layers of socio-ecological contextual information, including *interpersonal* and *ecological* language *dynamics*, using cross-disciplinary quantitative tools such as social network analysis and geospatial demographic analysis. Although these approaches have been discussed in the context of language acquisition (e.g., de Bot, 2008; de Bot et al., 2007), writing development (e.g., Baba & Nitta, 2014), foreign language anxiety (e.g., Mahmoodzadeh & Gkonou, 2015), irony comprehension (Tiv et al., 2023), and speech perception (Kutlu, 2020; Kutlu et al., 2022a, 2022b), other facets of the bilingual experience have not yet been considered. In this paper, we focus on bilinguals’ attitudes toward languages, which may be especially responsive to social-ecological influences.

Past work separately examined the influences of language background (e.g., Kircher, 2014), language use within close relationships (e.g., Karahan, 2004), sociocultural environments (e.g., Dörnyei & Csizér, 2002), and community infrastructure (Giles et al., 2006) on language attitudes (for a review of existing language attitudes frameworks, see Giles & Marlow, 2011). However, to our knowledge, social influences on language attitudes have not yet been explored systematically and simultaneously through a social-ecological framework. Moreover, a holistic view of language attitudes may have even greater importance within the context of bilingualism, as there may be greater individual variation and social influences that constrain bilingual than monolingual language use (Beatty-Martínez & Dussias, 2017; Beatty-Martínez et al., 2020; Bice & Kroll, 2019; Gullifer & Titone, 2020; Tiv et al., 2020). Accordingly, the present study bridges these gaps by applying a *Systems Framework of Bilingualism* (Titone & Tiv, 2023a; 2023b; Tiv, Kutlu, Gullifer, et al., 2022) to language attitudes of bilingual adults in a highly multilingual and multicultural setting. Specifically, we use the city of Montréal in Québec, Canada as a case study to investigate this systems approach to language attitudes, given the historical, political, and sociocultural context surrounding English, French, and multilingualism in the province.

## Systems framework of bilingualism

Similar to previous work (e.g., de Bot et al., 2007; Steffensen & Fill, 2014), the *Systems Framework of Bilingualism* (Titone & Tiv, 2023a; 2023b; Tiv, Kutlu, Gullifer, et al., 2022) suggests that people do not acquire language in a linear fashion—rather, language is much more intricate, complex, and interwoven into interdependent facets of the person and the environment. However, because traditional ecological-experimental methods have had difficulty quantifying the dynamics of language (Steffensen & Fill, 2014), social network analysis (reviewed in Scott, 2017; Tiv et al., 2020; Vitevitch, 2019) and geospatial demographic analysis (Tiv, Kutlu, Gullifer, et al., 2022) were jointly applied as a methodological approach that offered novel ways to measure the multilevel systems involved in language experience.

**Figure 1.**
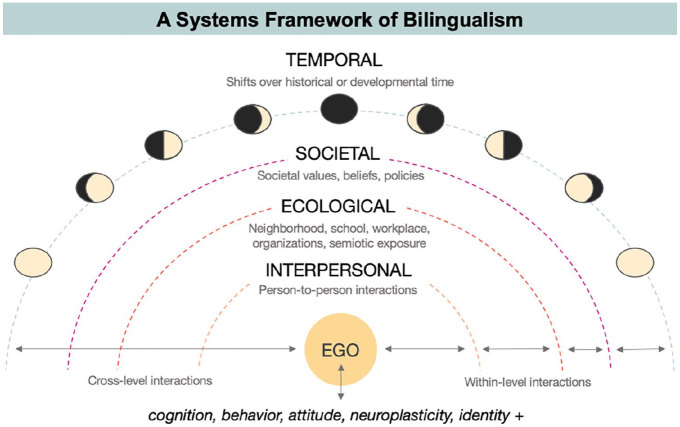
Systems framework of bilingualism (figure taken from Titone & Tiv, 2023b).

In this framework, an individual person is nested within a complex, dynamic, and interrelated system of linguistic influences (see Figure 1). First, *interpersonal language dynamics* consist of person-to-person interactions in different contexts of daily life. In our past work, we quantified interpersonal dynamics using language-tagged social networks (Titone & Tiv, 2023a; 2023b; Tiv, Kutlu, Gullifer, et al., 2022; Tiv, Kutlu, O’Regan, & Titone, 2022), which others have related to language use on Twitter (Eleta & Golbeck, 2014; Kim et al., 2014), language-related stress (Doucerain et al., 2015), and linguistic maintenance (Lanza & Svendsen, 2007). Second, *ecological language dynamics* include broader sources of ambient language exposure, such as individuals’ neighborhood, school, or workplace. To quantify this layer, our previous work used linguistic demographic data extracted from the Canadian Census (Titone & Tiv, 2023a; 2023b; Tiv, Kutlu, Gullifer, et al., 2022). Third, *societal language dynamics* refers to overarching characteristics of the society, such as the political and sociocultural contexts surrounding language in Québec. While we do not examine societal-level sources of sociolinguistic context in the present paper, other work from our group assesses these dynamics through cross-regional comparative analyses (Kutlu et al., 2022a; Tiv, Kutlu, O’Regan, & Titone, 2022). Finally, these systems influence one another while fluctuating over time. In the present work, we focus the scope of our analyses to interpersonal and ecological language dynamics.

## Language attitudes in Québec

### Theoretical background of language attitudes

Language attitudes refer to people’s evaluations of different aspects of language (Dragojevic et al., 2021; Giles & Ryan, 1982; Ryan et al., 1982). Previous research has proposed two evaluative dimensions underlying language attitudes: status and solidarity (Gardner & Lambert, 1972; Genesee, 2001; Genesee & Holobow, 1989; Ryan et al., 1982). The status dimension is associated with power, economic opportunity, and upward social mobility. As such, a language perceived as having high status is considered more prestigious and utilitarian than a language perceived as having low status (Kircher, 2022). However, the solidarity dimension is associated with personal identity and belongingness (Kircher, 2022). High solidarity with a language indicates that this language is linked to a person’s sense of self and sense of attachment to in-group members. According to social identity theory (Tajfel & Turner, 1986), individuals have a desire to achieve a positive social identity, leading them to positive biases favoring their in-group. Therefore, the greater an individual identifies with a given in-group, the higher they will evaluate their solidarity with the language spoken by that specific in-group. However, other work has also shown that this positive in-group bias may not be universal, such that individuals from minority groups may experience internalized stigma and thus rate their own group negatively (Hughes et al., 2015). In-group bias may also depend on other individual and contextual factors such as immigrant status or generation, which have been shown to play a role in code-switching among bilinguals (Chana & Romaine, 1984; Yim & Clément, in press). Furthermore, language attitudes, social identity, and social structures are inextricably intertwined—the differential power of social groups within a particular society are reflected in attitudes toward the languages spoken by these groups, which also in turn influence majority and minority language identity (e.g., Giles & Coupland, 1991; Giles et al., 1973; Riagáin, 2008; Ryan et al., 1982).

### Historical and political context of Québec

The historical, political, and sociocultural context surrounding English and French in Québec is sociolinguistically rich (Kircher, 2014; Leimgruber, 2020). While Québec’s provincial government’s official language is French, at the national level, Canada has two official languages: English and French. French speakers are concentrated in the province of Québec (~ 94%, Statistics Canada, 2017), where the maintenance of French dominance is largely attributed to sustained language planning efforts from the provincial government since the Quiet Revolution of the 1960s and 1970s (Bourhis, 1983). Indeed, until the 1970s, economic and industrial power mostly laid in the hands of the English-speaking minority in the city of Montréal, which is the urban center of the province (Hamers & Hummel, 1994; Kircher, 2022). Many historians have described the social climate of the early 1900s as “two solitudes”—the English-speaking minority occupying a large proportion of high-paying and high-status positions in the city, and the French-speaking majority remaining mostly in rural, religious communities (Hamers & Hummel, 1994; Kircher, 2014).

During the Quiet Revolution, French-speaking Québec underwent intense social and political changes, fueled by a growing sense of nationalism and reclamation of language and culture (Bourhis, 1983; Hamers & Hummel, 1994). The province’s legislative efforts to protect French language culminated into Bill 101 (i.e., the Charter of the French Language/Charte de la langue française), which implemented various pro-French legislation such as reinforcing French as the only official language of the province and requiring it to become the main language of the workplace, education, and public sphere (Kircher, 2014).

In addition to French and English, Québec, as well as Canada more broadly, is home to many Indigenous languages that pre-date English or French colonization, including many languages that are now extinct due to colonial forces (Khawaja, 2021). More recently, other languages, such as Arabic or Spanish, have also become entrenched in these regions through immigration. Specifically, in the city of Montréal, immigrants account for around 25% of the general population, with around the same proportion reporting “non-official languages” (i.e., other than French or English) as their mother tongue (Statistics Canada, 2017).

### Previous work on language attitudes in Québec

Given this historical and political context, many researchers have been interested in studying the language attitudes toward English and French in Québec. Moreover, with globalization and the influx of immigration in Montréal at the end of the 20th century, researchers have increasingly examined attitudes toward other languages, including those spoken by Indigenous, minority, and immigrant communities. Here, we will summarize the main findings of two eras of language attitudes research—before the implementation of Bill 101 (i.e., before 1977), and after its implementation (see Kircher, 2009, for a more comprehensive review).

While English speakers held economic power during the 1950s and 1960s, English was evaluated more positively by both English and French speakers, possibly mirroring global political shifts (e.g., World War II, globalization). More specifically, English speakers rated English more highly on the status dimension, while French speakers rated English more highly on both status and solidarity dimensions (Lambert et al., 1960; Preston, 1993). French speakers’ attitudes toward French might have been related to internalized stereotypes against—or sentiments connected to—their own group (Kircher, 2009). This could, for example, illustrate the dominance of English in both economic and social contexts.

After the introduction of Bill 101 in 1977, Francophones’ attitudes toward French on the solidarity dimension shifted to a more neutral or favorable stance (e.g., Bourhis, 1984; Genesee & Holobow, 1989; Kircher, 2014). However, both linguistic groups still favored English on the status dimension, although the status of French among Francophones increased (Genesee & Holobow, 1989; Kircher, 2009, 2014), suggesting the role of English as a global *lingua franca*. On the solidarity dimension, Laur (2001) found that both English- and French-speakers rated French very positively, hypothesizing that Quebecers perceived French as the public language and as “an integrative function in the Montréal community.” Through two experimental studies, Genesee and Bourhis (1982, 1988) also demonstrated that while French- and English-speaking Canadians expressed preference toward their own in-group language, language attitudes also had a sociostructural component linked to the relative position or status of language groups within social structure.

While some researchers have investigated attitudes toward languages other than English and French (e.g., Inuktitut; Taylor & Wright, 1989), there is a paucity of literature regarding attitudes toward multilingualism itself. More recently, Kircher and colleagues (2021) addressed this gap through an investigation of parents’ attitudes toward childhood multilingualism in Québec. Through a self-report questionnaire, the researchers revealed that three separate dimensions underpinned language attitudes: status and solidarity, as well as cognitive development which had not previously emerged in studies about individual languages. They also found that parents’ approaches to multilingualism and transmission of a heritage language were associated with parents’ attitudes toward multilingualism, whereas parent linguistic background and geographical location were not. Unknown from this work are the respective roles of proximal and distal language exposure on attitudes toward these languages and bi-/multilingualism more generally, as well as how these linguistic influences relate to each other, which brought us to carry out the present study.

## The present study

We investigated Montréal French–English bilinguals’ language attitudes through the *Systems Framework of Bilingualism* (Titone & Tiv, 2023a; 2023b; Tiv, Kutlu, Gullifer, et al., 2022). First, we took a bottom-up, data-driven approach to assess patterns underlying language attitudes by conducting a Principal Component Analysis (PCA). Second, we assessed the role of individual characteristics (i.e., first language) in language attitudes. Finally, we assessed the interrelated *interpersonal* and *ecological language dynamics* of bilinguals’ language attitudes through latent variables computed using language-tagged social network and census measures. Together, we suggest that this holistic social-ecological view of language attitudes offers a novel and comprehensive understanding of the social worlds of bilinguals in multilingual societies such as Montréal.

## Methods

### Participants

We recruited 123 participants aged 18–35 years old (*M* = 21.20, *SD* = 3.21) through the McGill Psychology participant pool and public advertising (i.e., social media posts and flyers written in both English and French) in the city of Montréal, Canada. The participants were compensated course credit and/or $10 per hour. The study was conducted at McGill University, where the language of instruction and administration is English.

Through a language history questionnaire (see Materials & Procedures), participants were asked to report the language they spoke in their first year of life. Participants reported acquiring their first language (L1) as English or English *and* another language (*n* = 32), French or French *and* another language (*n* = 56), both French/English or French/English *and* another language (i.e., simultaneous bilinguals, *n* = 35). Other languages included Arabic (2), Creole (3; 2 Haitian and 1 Mauritian), Croatian (1), Farsi (1), German (2), Italian (5), Khmer (1), Spanish (3), and Vietnamese (1). Participants also reported daily usage of these languages overall, with English being used at a higher proportion throughout the day (*M* = 63.36%, *SD* = 23.76) compared with French (*M* = 34.79%, *SD* = 23.13).

For gender, 82.11% self-identified as female (*n* = 101), 17.08% self-identified as male (*n* = 21), and 0.81% self-identified queer/non-binary (*n* = 1). Most participants grew up in a highly educated household, as 68.60% of parents/caretakers of our participants (*n* = 166) had a university degree. The ethnic background of the majority of the sample was White (*n* = 86, 69.92%). Other racial–ethnic backgrounds included Black (*n* = 6), East Asian (*n* = 1), Indigenous (*n* = 1), Latin American (*n* = 1), Middle Eastern (*n* = 4), Pacific Islander (*n* = 1), Southeast Asian (*n* = 1), and Other (*n* = 4). Several participants selected more than one background (*n* = 18, 14.63%). The majority reported being born in Canada (*n* = 80, 65.04%). Of those born in Canada, 70% (*n* = 56) reported being born in Québec and 62.5% (*n* = 35) of those born in Québec were born in Montréal. Those born outside of Canada reported living in Canada from 0 to 32 years (*M* = 6.33, *SD* = 7.80).

Given that our recruitment strategy included McGill University’s participant pool and public advertising through flyers posted on campus and in related social media groups, most participants were currently university students who reported their highest obtained education level being high school/secondary school (*n* = 39, 31.71%) or CEGEP/associate’s degree (*n* = 54, 43.90%). A smaller proportion reported university/college (*n* = 23, 18.70%) or Master’s/PhD (*n* = 6, 4.88%) as their highest obtained degree. While many participants reported attending primary school in French (*n* = 84, 68.29%), the majority reported attending university in English (*n* = 96, 78.05%). The distribution in high school seemed to be more equal, with 44.72% (*n* = 55) attending it in English and 54.47% (*n* = 67) in French.

### Materials and procedures

All materials and procedures were approved by the McGill University Research Ethics Board. Recruited participants were given two computer-based questionnaires in the lab. All participants (*n* = 123) completed a Language History and Attitudes Questionnaire. A subset of them (*n* = 73, 59.35%) completed a Social Network Survey (SNS), which was included in the experiment at a later phase. Both questionnaires were administered in person in English to ensure that all participants experienced the same treatment by researchers.

### Language History and Attitudes Questionnaire

Participants provided general and language-related demographic information. Then, they answered a series of 48 questions on their language attitudes toward English, French, language protection laws in Québec, Indigenous and minority languages, bilingualism, and multiculturalism. Response options were 7-point interval Likert-type scales (1 = *Totally disagree*, 7 = *Totally agree*). Several questions were adapted from Kircher’s (2009) Language Attitudes questionnaire (e.g., “French is a beautiful language”; see all items in Supplementary Materials). This questionnaire was administered with Qualtrics (Version May 2020; Qualtrics, 2020).

### Social Network Survey

Our survey is based on an egocentric network approach in which each respondent (also referred to as the “ego” in the domain of social networks) reports on individuals they know (referred to as “alters” in network terminology). These alters make up the nodes in the network, and relationships between the alters make up the ties between nodes. Additional information regarding these social network terms is available in Appendix 1.

As such, participants (i.e., egos) were asked to nominate eight to twelve people (i.e., alters) with whom they have regularly interacted over the past 6 months across various communicative contexts (i.e., home, work, school, social, and every day). Next, they answered basic demographic questions about each of these people. Most relevant to our research questions, respondents also indicated which language or languages they used with each alter. Finally, participants indicated which alters interacted with each other, and this information formed the ties between alters. The SNS was created and administered with the Network Canvas software (Version 5.1.0; Complex Data Collective, 2016).

**Figure 2.**
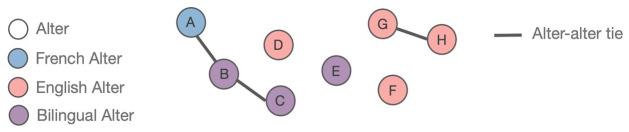
Example of a language-tagged egocentric network. In this toy network, the respondent or ego themselves is not shown, and nodes A through H are all alters. Edges A-B, B-C, and G-H are alter-alter ties, meaning that, for example, the respondent perceives alters A and B to interact with one another. For the purposes of our study, we also tagged each alter with the language(s) the respondent uses with them to create language-tagged subnetworks. In the illustration, the full network comprises all eight alters; the English subnetwork comprises alters D, F, G, and H; the French subnetwork comprises alter A; and the Bilingual subnetwork comprises alters B, C, and E.

Using participants’ full personal network, we created *language-tagged subnetworks* (English, French, and Bilingual; see Figure 2 for illustrative example). We included all alters who interacted with the respondent using at least French or English. This definition was both specific enough to detect subtle differences around language usage and general enough to render sufficiently large subnetworks for extracting meaningful structural and compositional network measures. As such, the English subnetwork included all alters who only use English or English *and* another language (other than French) with the respondent, while the French subnetwork included all alters who only use French or French *and* another language (other than English) with the respondent. The Bilingual subnetwork contained the alters who use both English and French with the respondent. From these subnetworks, we computed several network measures which either reflected characteristics of the alters or the relationships among alters, using the *igraph* package (Csardi & Nepusz, 2006) in R (R Development Core Team, 2019). Here, we focus on the five measures included in the *Systems Framework of Bilingualism* (Titone & Tiv, 2023a; 2023b; Tiv, Kutlu, Gullifer, et al., 2022), which were chosen because they captured unique aspects of the network structure while demonstrating distributional variance.

#### Network size

Network size refers to the total number of nodes in a network, or the total number of alters nominated by one participant. Since we divided the whole network into language-specific subnetworks, each subnetwork size reflects the amount of exposure the respondent has to each language group. For instance, the English subnetwork’s network size would refer to the number of alters who minimally use English (i.e., English or English plus another language) with the respondent. In the toy network shown in Figure 2, the total network size would be 8, whereas the English subnetwork’s network size would be 4.

#### Network density

Network density reflects the overall interconnectedness of the people in the network. This metric is computed from the proportion of the total number of ties divided by the maximum number of possible ties. This measure ranges from 0 to 1, with 1 indicating that alters know all other alters in the network and 0 indicating that no alters know each other. In the context of language-based social networks, network density may reveal information about the quality of exposure to each language (i.e., whether exposure to English comes from unconnected people or from a group of connected people).

#### Number of components

This network measure refers to the number of subgraphs (i.e., clusters of connected nodes) in a network. A network that is fully connected (i.e., every tie is connected to one another) would have only one component, whereas a network in which no alters know each other would have the same number of components as alters. For example, the toy network shown in Figure 2 would have five components.

#### Network strength

Network strength reveals some aspect of the relationships between alters, which in this case focused on the perceived closeness between alters. This measure was calculated using the sum of all adjacent ties weighted by the respondent’s perceived closeness between the alters in their network. As such, this measure reflects the overall closeness of the alters in a network.

#### Eigenvalue centrality

Eigenvalue centrality assesses the extent to which important or well-connected nodes are also linked with important nodes in a network. In other words, this measure assesses the overall quality of relationships between alters in a network. The linguistic characteristics of highly central alters may reveal how central certain languages are to the respondent’s daily life.

### Canadian Census survey

Using the 2016 Canadian Census Profile (catalog number: 98-401-X2016046; Statistics Canada, 2016), we assessed language use in our sample’s residential neighborhoods. Three measures were included in Tiv et al. (2022) *Systems Framework of Bilingualism*: English Index, French Index, and Language Diversity. All measures were calculated from the first three digits of each respondent’s residential postal code (i.e., Forward Sortation Area).

#### English Index

English Index is a continuous metric of the prevalence of English in each regional area. This measure was calculated by dividing the total number of respondents who reported English as their first language or as one of their first languages by the total number of respondents in a Forward Sortation Area. As such, the English Index was a proportion ranging from 0 to 1, such that a higher score implied a higher number of people in a neighborhood with English as their first language, while a lower score implied a lower number of people with English as their first language.

#### French Index

Similar to the English Index, the French Index is a continuous metric of the prevalence of French in each regional area. This measure was also calculated by dividing the total number of respondents who reported French as their first language or as one of their first languages by the total number of respondents in a Forward Sortation Area.

#### Language Diversity

The Language Diversity of a residential neighborhood was computed using the Index of Qualitative Variation, which is a measure of variability or diversity within nominal variables (see Gullifer & Titone, 2020, for a conceptually similar characterization of people’s individual language experiences in terms of *language entropy*). It is based on the ratio of the total number of observed differences to the maximum number of possible differences within a distribution. This measure varies between 0 and 1, with 0 indicating no variation/diversity and 1 indicating high variation/diversity.

Wilcox (1973) proposed six indices that can be used to measure qualitative variation, which have been implemented in R with the *qualvar* package (Gombin, 2018). For our analyses, we used Deviation from the Mode, a standardized variation ratio that “can be thought of as an index of deviation from the modal frequency, analogous to the variance as a measure of deviation from the mean” (Wilcox, 1973). The formula is stated below:

$$1-\frac{\sum_{i=1}^{k}(f_{m}-f_{i})}{N(K-1)},$$

where *f_i_* is the frequency of the *i*th category, *f_m_* is the frequency of the modal category, *N* is the number of cases, and *K* is the number of categories.

## Results

### Components of Language Attitudes Scale

To assess patterns underlying the responses to our language attitude scale, and to decompose the large number of variables into smaller subscales, we took a bottom-up, data-driven approach by conducting a PCA on 48 language attitude items using the full sample (*n* = 123). PCA is a descriptive statistical technique that reduces the dimensionality of a dataset by transforming values into new uncorrelated variables that maximize variance (Jain & Shandliya, 2013; Jolliffe & Cadima, 2016). For our analyses, we conducted PCA with an oblimin rotation on polychoric correlations, which are an alternative to Pearson product-moment correlations more suitable for discrete data, like Likert-type scales (Bialystok, 2001; Kolenikov & Angeles, 2004; Olsson, 1979). Bartlett’s Test of Sphericity (Bartlett, 1954) and the Kaiser–Meyer–Olkin (Cerny & Kaiser, 1977) index revealed that our data was suitable for PCA (Supplementary Materials).

### Results of PCA

The results of parallel analysis conducted with the “fa.parallel” function in the *psych* R package (R Development Core Team, 2019; Revelle, 2021) revealed four to five principal components. Given the fifth component laid very close to the random eigenvalues and was theoretically meaningful, we opted for a five-component solution (see Supplementary Materials for analyses with a four-component solution that omitted this component in which all findings remained consistent). Here, we report the percentage of variance accounted for by each component, which should be taken into consideration when interpreting the results of our analyses. The correlation matrix confirms that the obliquely rotated solution was adequate, given the correlations between the components. Five principal components collectively accounted for 46.83% of the total variance. See Supplementary Materials for the parallel analysis scree plot, correlation matrix table, and full-component loadings.

The first component (PC1; 18.63% of total variance) comprised items that were related to personal identity and feelings of belongingness toward French and away from English, such as “French makes me feel secure” (0.71) and “I feel true to myself when I speak French” (0.71). In contrast, items related to solidarity toward English, such as “I prefer to speak English to comfort someone” (−0.65), were highly negatively loaded. Thus, because French items loaded positively and English items loaded negatively on the solidarity dimension, we named this component *French* *>* *English Solidarity*.

The second component (PC2; 13.93% of total variance) grouped general attitudes toward English, as well as items that related to both solidarity and status of English. Highly positively loading items included “English is a beautiful language” (0.86), “English is a language that is well-suited for modern society” (0.69), and “Knowing English is an important part of my personal identity” (0.61). Items that loaded negatively were relatively weakly loaded, such as “French is more elegant than English” (−0.29). Thus, we labeled this component *English General*.

The third component (5.55% of total variance) included high loading items such as “I believe there should be more legislation protecting Indigenous languages of Canada” (0.74) and “I believe there should be more legislation protecting the languages spoken by immigrants in Canada” (0.66). This component also included weakly negatively loading items such as “English is more elegant than French” (−0.31). Therefore, because the high loading items related to attitudes toward the protection of Indigenous and immigrant languages, we named this component *Minority Language Protection.*

The fourth component (4.74% of total variance) related to the perceived prestige (i.e., an aspect or attribute of overall status) of both French and English, such that “Speaking in French increases the value and prestige of what I say” (0.65) and “Speaking in English increases the value and prestige of what I say” (0.54) were both positively loaded. This component also seemed to capture attitudes toward the elegance of French over English, with “French is more elegant than English” (0.59) loading positively and “English is more elegant than French” (−0.37) loading negatively. Thus, we named this component *French&English Prestige*.

Finally, the fifth component (3.99% of total variance) grouped items related to the protection of French within Québec and within the greater Canadian cultural heritage, such as “I think more legislation should be put in place to protect the vitality of the French language in Quebec” (0.65) and “Knowing French is a significant part of Canadian cultural heritage” (0.61). Because negatively loading items included attitudes toward the protection of English in Québec (−0.22), we labeled this component *French Language Protection*.

### Characteristics of the respondent (L1)

First, we investigated the associations between a respondent’s individual characteristics and their language attitudes (i.e., no social network or neighborhood characteristics). Specifically, we examined the role of first language (L1) by dividing the full sample (*N* = 123) into three groups on the basis of their first language—English or English plus another language (*n* = 32), French or French plus another language (*n* = 56), and French/English or French/English plus another language (i.e., simultaneous bilinguals; *n* = 35).

**Figure 3.**
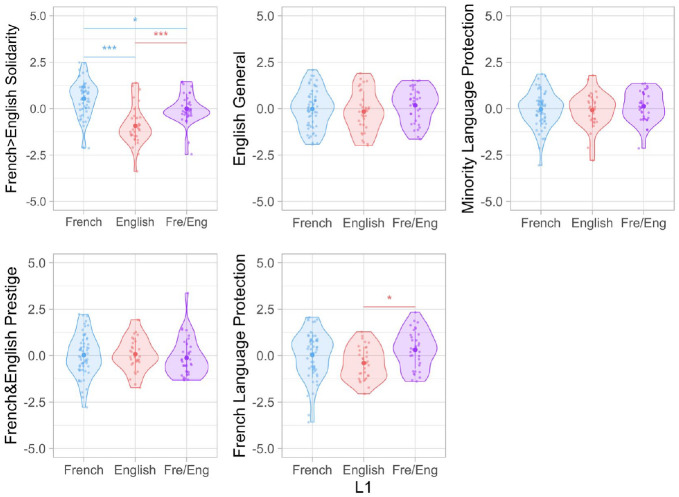
Mean component scores for L1 groups. *Note.* Error bars indicate plus or minus one standard error of the mean. ***p < .001; **p < .01; *p < .05.

For each of the five components extracted by PCA, we fit a simple linear regression with L1 (i.e., English, French, and French/English) as the independent variable and component scores as the dependent variable (see Figure 3 and Table 1). We found that L1 was a significant predictor of scores on *French* *>* *English Solidarity* (PC1, *F*(2) = 23.10, *p* < .001) and *French Language Protection* (PC5, *F*(2) = 3.62, *p* = .03).

**Table 1.** Mean component scores for L1 groups.

L1(*N* = 123)		Principal component				
		French > English Solidarity (PC1)	English General (PC2)	Minority Language Protection (PC3)	French > English Prestige (PC4)	French Language Protection (PC5)
French (*n* = 56)	Mean	0.53	−0.02	−0.03	0.03	0.04
	*SE*	0.14	0.14	0.13	0.15	0.17
English (*n* = 32)	Mean	−0.93	−0.16	−0.08	0.08	−0.40
	*SE*	0.18	0.20	0.17	0.16	0.16
French/English (*n* = 35)	Mean	−0.00	0.17	0.13	−0.12	0.31
	*SE*	0.14	0.16	0.14	0.17	0.17
	*Df*	2	2	2	2	2
	*F-value*	23.10	0.91	0.50	0.34	3.62
	*p-value*	**< .001**	.41	.61	.72	.**03**

*Note.* Bold = significant at *p* < .05.

Tukey’s honest significant difference (HSD) post hoc tests revealed that the L1 French and L1 French/English groups had significantly higher scores on *French* *>* *English Solidarity* compared with the L1 English group (English vs French HSD = 1.46, *p* < .001; English vs French/English HSD = 0.93, *p* < .001). Moreover, the L1 French group had the highest scores among all L1 groups (French vs French/English HSD = −0.53, *p* = .03). This indicates that individuals who learned French in their first year of life expressed significantly more positive solidarity attitudes (related to personal identity and belongingness) toward French than other L1 groups, whereas individuals who learned English in their first year of life expressed significantly more positive solidarity attitudes toward English than other L1 groups.

Moreover, Tukey’s HSD post hoc tests also revealed that the L1 French/English group had significantly higher scores on *French Language Protection* than the L1 English group (HSD = 0.710, *p* = .02), while the L1 French group did not differ from other L1 groups. No other pairwise differences were significant.

### Interpersonal and ecological language dynamics

Based on the *Systems Framework of Bilingualism* (Titone & Tiv, 2023a; 2023b; Tiv, Kutlu, Gullifer, et al., 2022), we examined the interpersonal and ecological language dynamics of bilinguals’ language attitudes. Here, we used latent variables computed by Tiv et al. (2022), which were extracted through an exploratory factor analysis (EFA) conducted on 18 normalized variables. To assess the interpersonal layer of linguistic influence (i.e., person-to-person interactions), five social network variables computed from respondents’ SNS were included (i.e., network size, density, number of components, Eigencentrality, strength) for each of the three language-tagged subnetworks (i.e., English, French, English/French Bilingual). To assess the ecological layer (i.e., neighborhood-level language exposure), three indices from the 2016 Canadian Census (Statistics Canada, 2016) were included: English Index, French Index, and Language Diversity.

Four factors were extracted from the EFA: *French Network* (on which all five French network measures loaded positively), *English Network* (on which all English network measures loaded positively), *Bilingual Network* (on which all bilingual network measures loaded positively), and *Ecology* (on which Language Diversity and English Index loaded positively while French Index loaded negatively). See Tiv and colleagues (in press) for full factor loadings and factor correlation table. The associations between respondents’ L1 and these four latent factors are shown in Supplementary Materials.

### Predicting language attitudes

**Figure 4.**
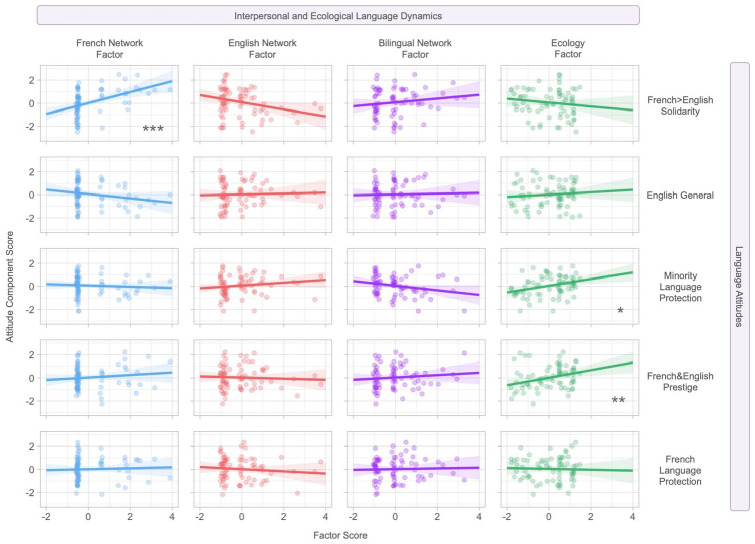
Robust multiple linear regressions predicting language attitudes component scores. *Note.* Shaded area represents plus or minus one standard error of the mean. ***p < .001; **p < .01; *p < .05.

We examined whether the five components of language attitudes (i.e., *French* *>* *English Solidarity, English General, Minority Language Protection, French&English Prestige, French Language Protection*) were predicted by the four latent variables of the *Systems Framework* (i.e., *French Network, English Network, Bilingual Network, Ecology*) (see Figure 4 and Table 2). To account for potential outliers while capturing the diversity present in bilinguals’ social networks, we conducted robust linear regressions, which minimize the weight of single data points with large residuals. All models were conducted using the *MASS* (Venables & Ripley, 2002) package in R, and subsequent robust F-tests were computed using the *sfsmisc* (Maechler, 2021) package. All variables included in our models were treated continuously.

**Table 2.** Robust multiple linear regressions predicting language attitude component scores.

Predictor	French > English solidarity						English general						Minority language protection					
	*B*	*SE B*	β	95% CI	*t*	*p*	*B*	*SE B*	β	95% CI	*t*	*p*	*B*	*SE B*	β	95% CI	*t*	*p*
French factor	0.48	0.14	.42	[0.21, 0.75]	3.51	**< .001**	−0.20	0.12	−0.22	[−0.44, 0.05]	−1.60	.12	−0.03	0.10	−0.04	[−0.23, 0.16]	−0.35	.73
English factor	−0.16	0.14	−.14	[−0.44, 0.11]	−1.18	.24	−0.02	0.13	−0.02	[−0.27, 0.23]	−0.15	.88	0.06	0.10	0.07	[−0.14, 0.25]	0.57	.57
Bilingual factor	0.24	0.14	.20	[−0.04, 0.52]	1.73	.09	0.00	0.13	0.00	[−0.26, 0.25]	−0.02	.99	−0.19	0.10	−0.23	[−0.39, 0.00]	−1.95	.06
Ecology	0.00	0.15	.00	[−0.29, 0.30]	0.02	.99	−0.01	0.13	−0.01	[−0.28, 0.26]	−0.07	.95	0.24	0.10	0.28	[0.03, 0.45]	2.29	.**03**
*R* ^2^				.25						.04						.14		

	French & English Prestige						French Language Protection					
Predictor	*B*	*SE B*	β	95% CI	*t*	*p*	*B*	*SE B*	β	95% CI	*t*	*p*
French Factor	0.17	0.11	.19	[−0.05, 0.38]	1.51	.14	0.03	0.12	0.03	[−0.22, 0.27]	0.22	.83
English factor	−0.03	0.11	−.03	[−0.25, 0.20]	−0.24	.81	−0.09	0.13	−0.10	[−0.34, 0.16]	−0.72	.48
Bilingual factor	0.12	0.11	.12	[−0.11, 0.34]	1.05	.30	0.03	0.13	0.03	[−0.22, 0.28]	0.23	.82
Ecology	0.40	0.12	.40	[0.17, 0.64]	3.40	.**001**	0.03	0.13	0.03	[−0.23, 0.30]	0.25	.80
*R* ^2^				.11						.01		

*Note.* Bold = significant at *p* < .05. CI = confidence interval.

First, the *French Network* factor significantly predicted scores on *French* *>* *English Solidarity* (PC1; *B* = 0.48, *SE B* = 0.14, β = .42, 95% CI = [0.21, 0.75], *t* = 3.51, *p* < .001). These results suggest that having a larger, more interconnected, and strongly influential French-speaking personal network was associated with more positive attitudes toward French as part of their personal identity.

Next, our analyses showed that the *English Network* factor did not significantly predict any language attitude components. This suggests that the size, interconnectedness, and centrality of a respondent’s English-speaking personal network did not play a role in their language attitudes. Similarly, we found that the *Bilingual Network* also did not significantly predict any language attitude components, suggesting that the size, interconnectedness, and centrality of a respondent’s bilingual personal network was not associated with their language attitudes.

Finally, our analyses revealed that the *Ecology* factor significantly predicted scores on *Minority Language Protection* (PC3; *B* = 0.24, *SE B* = 0.10, β = .28, 95% CI = [0.03, 0.45], *t* = 2.29, *p* = .03) and *French&English Prestige* (PC4; *B* = 0.40, *SE B* = 0.12, β = .40, 95% CI = [0.17, 0.64], *t* = 3.40, *p* = .001). This suggests that higher neighborhood-level diversity and English use was associated with more positive attitudes toward the protection of minority languages. Similarly, higher neighborhood-level linguistic diversity and English use was also associated with more positive attitudes toward the perception of both English and French as prestigious languages.

Our models showed that no predictors were significantly associated with the *English General* (PC2) and *French Language Protection* (PC5) attitude components. It is also important to note that both components only accounted for a minimal amount of variance in our data (*R*^2^ = .04 for *English General* and *R*^2^ = .01 for *French Language Protection*). Relatedly, across all models, our model of *French* *>* *English Solidarity* (PC1) fit the best, as it accounted for the largest percentage of variance in our data (*R*^2^ = .25).

## Discussion

The primary goal of this study was to examine the *interpersonal* and *ecological language dynamics* of language attitudes among bilingual adults, through the lens of the *Systems Framework of Bilingualism* (Titone & Tiv, 2023a; 2023b; Tiv, Kutlu, Gullifer, et al., 2022). We conducted this investigation in a highly multilingual and multicultural setting, using the city of Montréal as a case study. First, we took a bottom-up, data-driven approach to assess patterns underlying language attitudes. Second, we assessed the role of individual characteristics (i.e., first language) in language attitudes. Finally, we combined language-tagged social network analysis and geospatial demographic analysis as a novel methodological approach to quantify the interrelated systems involved in bilinguals’ attitudes toward languages.

### Components of language attitudes

Consistent with past findings on the evaluative dimensions of language attitudes (e.g., Genesee, 2001; Genesee & Holobow, 1989), one of the components highlighted by our PCA related to the solidarity dimension. In particular, component loadings of *French* *>* *English Solidarity* (PC1) clearly patterned along two poles—positively loading on feelings of identity, belongingness and comfort toward French, and negatively loading on solidarity feelings toward English. However, unlike previous findings, our analysis did not uncover the status dimension as clearly. In fact, rather than loading on the same component, items related to English status seem to fragment into two separate subscales—a utilitarian aspect, associated with general attitudes toward English (i.e., *English General*, PC2), and a prestige aspect, associated with the perceived prestige of both English and French (i.e., *French&English Prestige*, PC4). In other words, our results suggest that bilinguals evaluate the status of English using two distinct aspects—its utility for modern society and its perceived prestige.

While previous work on language attitudes considered solidarity and status as independent dimensions (e.g., Genesee, 2001; Genesee & Holobow, 1989; Kircher, 2014), our results suggest that bilinguals evaluate English in a more integrative way compared with French, such that attitudes toward several aspects of English (i.e., general, solidarity and utilitarian status) were grouped together on the *English General* (PC2) component. This might reflect increasing globalization and internalization of an international youth identity in the current social climate, as well as the domination of English as the global *lingua franca* (Kircher, 2014, 2022). Collectively, this could result in the concurrence of English identity and English utility, such that the feeling of belongingness to English as a global language was also associated with the feeling that English is important for modern society. However, it is also important to note that the present sample consisted primarily of students from English-speaking universities, which could explain why some patterns of language attitudes were inconsistent with prior work.

### Characteristics of the respondent

In line with previous research on language attitudes after the implementation of language policies protecting French in Québec such as Bill 101 (e.g., Kircher, 2014), L1 French speakers rated French more positively on the solidarity dimension than L1 English speakers and simultaneous bilinguals, whereas L1 English speakers rated English more positively than other L1 groups. This finding also builds on existing evidence of social identity theory (Tajfel & Turner, 1986), where higher identification with an in-group leads to higher attribution of solidarity toward the language spoken by that in-group. While recent work demonstrated that the in-group or reference group may relate to broader elements of linguistic identity or community-level subgroups (e.g., frequent code-switchers, other bilingual biculturals) in Cantonese-English bilinguals (Yim & Clément, 2019, 2021), our findings suggest that the in-group with which participants identified can also relate to others who share their first language. In addition, we did not find any evidence of internalized in-group stigma (Hughes et al., 2015), such that the relative minority status of English within the French-dominant province of Québec could be offset by the role of English as a global *lingua franca*, and vice versa.

Moreover, ratings on the perceived prestige of both languages did not differ significantly between L1 groups. This finding contrasts with research conducted before the implementation of language policies protecting French in Québec in the 1970s, which suggested that attitudes toward the status of a language differs depending on the L1 (e.g., Genesee & Holobow, 1989). L1 French speakers also rated attitudes toward the protection of French more positively than simultaneous bilinguals. These results highlight the role of individual differences in first language, and may also indirectly reflect the historical, political, and sociocultural influences stemming from the implementation of language planning policies. Finally, L1 groups did not differ in their attitudes toward English generally or toward the protection of minority languages, suggesting the overall importance of English and openness to diverse languages/cultures among young English–French bilinguals in Montréal.

While we only considered L1 among other individual characteristics of respondents, we want to call attention to a shift in paradigm in how L1 is conceptualized compared with previous research. We believe that the L1 experience cannot be separated from lived experiences of language users, such that L1 represents not only the first language of a respondent but a collection of ideological manifestation of using L1 which may or may not be inclusive of L2 or LX experiences. This shift in paradigm highlights the intersectionality of bilingualism research with language attitudes. Importantly, this is where the Systems Framework of Bilingualism (Titone & Tiv, 2023a; 2023b; Tiv, Kutlu, Gullifer, et al., 2022) is able to bring cutting edge interpretations—it is not treating any of the linguistic layers of influence as being simple or redundant, but each layer as being unique, interconnected, and context-dependent.

### Interpersonal and ecological language dynamics

Most importantly, our results showed that interpersonal and ecological linguistic influences, as measured by language-tagged social networks and neighborhood-level language patterns, respectively, jointly predicted bilinguals’ attitudes toward languages. In particular, having a larger, more interconnected, and influential French-speaking personal network was associated with more positive solidarity attitudes toward French among all English–French bilinguals. This finding relates to previous research in social psychology showing that people in social networks that share more similar views (i.e., echo chambers) hold stronger attitudes than those in social networks that have less similar views (e.g., Dalege et al., 2019; Levitan & Visser, 2008, 2009). For instance, when nested in a larger, more interconnected, and strongly influential personal network of French speakers, the respondent is surrounded by an immediate environment that is more linguistically homogeneous than the environment of a respondent with a smaller, less interconnected, and weakly influential personal network of French speakers. Thus, this could explain why the respondent holds stronger and more positive attitudes toward French—a language congruent with the alters that make up the French subnetwork.

Curiously, we did not observe the corresponding effect for English, such that having a larger, more interconnected, and influential English-speaking personal network was *not* associated with more positive attitudes toward English. Indeed, a respondent’s English subnetwork was not associated with any components of language attitudes. Because the bilinguals in our sample evaluated English more integratively than French, as shown by our PCA results, we conducted additional top-down analyses by isolating the specific questions used by Kircher (2009) to analyze the general, solidarity, and status dimensions of English and French (Supplementary Materials). In other words, we aimed to test whether our data-driven approach was in line with past theoretically-based approaches in language attitudes. We found that the results of both approaches were consistent with one another, suggesting that the role of English as the global *lingua franca* may supersede the possible echo chamber effects found in the French personal networks (e.g., Dalege et al., 2019; Levitan & Visser, 2008, 2009). That is, because of the status of English as the *lingua franca* of modern society, a respondent nested in a larger, more interconnected, and strongly influential English-speaking personal network may not necessarily hold more positive attitudes toward the language congruent with people that make up their network (i.e., English).

Importantly, our novel quantifications of sociolinguistic context revealed subtle dynamics in bilinguals’ language attitudes that were undetected by the conventional, first language analysis. Indeed, we found that ecological language dynamics also played important roles in the language attitudes of bilinguals. Particularly, higher neighborhood-level linguistic diversity and English use was associated with more positive attitudes toward the prestige of both French and English. Furthermore, higher neighborhood-level linguistic diversity and use of English was also associated with more positive attitudes toward the protection of minority languages. This finding is in line with extensive research showing that intergroup contact—though not only direct, person-to-person contact but also indirect or vicarious contact—reduces intergroup bias and increases positive attitudes toward out-group members (see Dovidio et al., 2017, for a review). In the current study, the presence of higher linguistic diversity within an individual’s neighborhood may lead to increases in both direct and indirect contact with minority languages (i.e., Indigenous and immigrant languages), resulting in more positive attitudes toward and awareness of these languages and the protection of their vitality within Canada.

Relatedly, an important application of the social-ecological framework has been in the study of prejudice, or negative attitudes toward out-group individuals based on a certain trait (Hinner, 2020). In particular, recent studies have looked at how social-ecological theory may inform policies and interventions for reducing prejudice and fostering diversity in education and in the workplace (e.g., Bond & Haynes, 2014; McKown, 2005). Because these attitudes are influenced by interactions with others and maintained by broader social structures, a holistic approach may be better able to capture this complex interplay of systems. Thus, future work should examine language attitudes through an in-group/out-group point of view, while keeping in mind the *Systems Framework* (Titone & Tiv, 2023a; 2023b; Tiv, Kutlu, Gullifer, et al., 2022).

### Limitations and future directions

We acknowledge several limitations in this present work. To start, we note the vulnerability of questionnaire study designs to the effects of social desirability bias (i.e., answering in a way that is perceived more favorably by others), especially when it comes to eliciting language attitudes (Kircher, 2014, 2022). Given that participants completed the study in an English-dominant context (e.g., being in a laboratory in an English university, reading English materials), social desirability is also important to consider in relation to language attitudes toward English. Yet, we believe the relative anonymity offered by computer-administered surveys compared with experimenter-administered interviews minimizes the risk of socially desirable answers. Nonetheless, future work may explore bilinguals’ indirect attitudes toward languages through methods such as a matched-guise design (Kircher, 2014) or an adapted Implicit Association Test (Pantos & Perkins, 2013).

Next, the subjective nature of egocentric network approaches may affect the reliability of our data. Indeed, because all information about each alter’s background, language use and relationships with other alters are filtered through the lens of the participant, this data is vulnerable to individual recall bias as well as time decay (Marsden, 1990). As mentioned previously, participants also completed the study in an English-dominant context, possibly leading to increased cuing of alters with whom they interact in English. One way to mitigate this limitation could be to utilize snowballing techniques (i.e., nominated alters are subsequently contacted and recruited for participation) or to increase the number of alters elicited, which could be directions for future research. Here, we focused on language use between the respondent and each alter, rather than between alters, which likely increased the reliability of the responses.

Another important limitation concerns the correlational nature of our study, preventing inferences about the causality of the role of individual characteristics (i.e., first language), social networks, and broader environmental influences on language attitudes. Indeed, although our results demonstrate associations between these factors, directionality cannot be established. For instance, apart from the interpersonal and ecological linguistic influences investigated here, it is equally possible that individuals with a priori attitudes toward English or French choose the people and the languages they want to interact with. In all likelihood, these relationships may to some extent be bidirectional, given the interrelated nature of social and cognitive processes (Smith et al., 2020).

Finally, our sample mostly consisted of university students recruited from English-speaking institutions, which may affect the generalizability of our results. Moreover, because student populations often have changes in housing, the validity of our results concerning ecological language dynamics, which are dependent on residential postal code, may be affected. Relatedly, among the respondents born outside of Canada, there was a wide range in duration of residence within Montreal. While we decided to retain all participants to better reflect the meaningful heterogeneity that exists among Montreal bilinguals, there may be differences in language attitudes based on length of stay. Future research should also consider including more diverse populations, such as allophones, first- and second-generation immigrants, and other age groups (e.g., youth, older adults), who can provide higher generalizability and additional insights into language attitudes within multicultural settings. Furthermore, given differences in language policies in other cities or provinces (i.e., societal layer in *Systems Framework*) that also have different colonial and linguistic histories (i.e., temporal layer), we may find other patterns of language attitudes that may be meaningful directions for future research.

With these caveats in mind, the results of this study nevertheless reinforce a holistic understanding the interpersonal and ecological language dynamics of language attitudes in bilingual adults living in multilingual settings, using the city of Montréal as a case study. Moreover, the findings bolster the idea that individuals are influenced by smaller- to larger-scale systems that interact and influence one another. This systems approach to bilingualism offers novel insights into aspects of bilinguals’ experiences, such as language attitudes, that conventional psycholinguistic approaches cannot provide. A more holistic perspective on language attitudes, combined with the cutting-edge quantitative tools offered by network science and geospatial demographic analysis, can have important implications for future language planning policies in multicultural societies.

## Supplemental Material

sj-docx-1-ijb-10.1177_13670069221133305 – Supplemental material for A systems approach to multilingual language attitudes: A case study of Montréal, Québec, Canada Supplemental material, sj-docx-1-ijb-10.1177_13670069221133305 for A systems approach to multilingual language attitudes: A case study of Montréal, Québec, Canada by Ruo Ying Feng, Mehrgol Tiv, Ethan Kutlu, Jason W. Gullifer, Pauline Palma, Elisabeth O’Regan, Naomi Vingron, Marina M. Doucerain and Debra Titone in International Journal of Bilingualism

## Appendix 1

### Fundamentals of social network analysis

Social network analysis is an application of network and graph theory that examines the relationships and flow of information among social entities (e.g., people, groups, communities). It has been used to study various aspects of the bilingual experience like language brokering (Kim et al., 2014), communication-related acculturative stress (Doucerain et al., 2015), and communicative patterns (Tiv et al., 2020), with the overall aim to understand complex systems. In this section, we review the basic fundamental terms and concepts in social network analysis (see Hawe et al., 2004; Scott, 2017; Vitevitch, 2019; Wasserman & Faust, 1994 for more comprehensive reviews).

### Basic elements of a network

See Figure 2 in the main manuscript for an example of a network.

#### Node

A node, also referred to as vertex or actor, is an element or entity in a network. For example, when examining the patterns of communication within a social group, nodes represent each of the individuals within that group. In the toy network displayed in Figure 2, each circle (e.g., A, B, C) represents a node, or a person.

#### Edge

Also called tie or link, an edge is an interaction or a relationship between two nodes. For instance, in the example mentioned above, an edge is formed when two individuals communicate with each other. In Figure 2, the lines between nodes A-B, B-C, and G-H are all edges in the network.

### Types of networks

We can broadly classify networks into two types. *Sociocentric or complete networks* include all possible ties within a broad social structure (e.g., group, community). However, *egocentric or personal networks* look at relationships from a single node’s point of view. Due to the relative ease of data collection and wide availability of standardized questionnaire formats, egocentric network approaches are more commonly used in sociological and psychological research (Wasserman & Faust, 1994). These networks are composed of connections between a focal node (i.e., the ego or the respondent) and the other nodes in its network (i.e., alters or the people named by the respondent), as well as connections between alters from the ego’s perspective (i.e., alter-alter ties). Figure 2 displays an example of an egocentric network.
