# Supplementary material for: A systems approach to multilingual language attitudes: A case study of Montréal, Québec, Canada
Source: Int J Billing. 2023 Apr 25;28(3):454–78. doi: 10.1177/13670069221133305 (PMC11178480; doi:10.1177/13670069221133305)
Supplement: sj-docx-1-ijb-10.1177_13670069221133305 – Supplemental material for A systems approach to multilingual language attitudes: A case study of Montréal, Québec, Canada [file sj-docx-1-ijb-10.1177_13670069221133305.docx]

**Supplementary Materials**

*A Systems Approach to Multilingual Language Attitudes: A Case Study of Montréal, Québec, Canada*

Table of contents

[A. Language Attitudes Questions in LHQ 2](#_Toc93960331)

[B. Results of Principle Components Analysis 5](#_Toc93960332)

[Figure B1 5](#_Toc93960333)

[Table B1 6](#_Toc93960334)

[Table B2 7](#_Toc93960335)

[C. Associations Between L1 and Latent Factors 10](#_Toc93960336)

[Table C1 10](#_Toc93960337)

[D. Predicting Language Attitudes Based on Predefined Categories 11](#_Toc93960338)

[Table D1 11](#_Toc93960339)

[Table D2 13](#_Toc93960340)

[Table D3 14](#_Toc93960341)

[Table D4 15](#_Toc93960342)

[E. Results for Four Components 16](#_Toc93960343)

[Table E1 16](#_Toc93960344)

[Table E2 19](#_Toc93960345)

[Table E3 20](#_Toc93960346)

[F. References 21](#_Toc93960347)

# A. Language Attitudes Questions in LHQ

Bold = items taken from Kircher (2009)

1. I am comfortable with English currently being the global language.
2. I value bilingualism.
3. I believe that people can have multiple identities at the same time. (E.g. Black + Canadian + Quebecer + English-speaking).
4. I support Bill 101.
5. I agree with the laws protecting the French language in Quebec.
6. I believe there should be more legislation protecting Indigenous languages of Canada.
7. I think more legislation should be put in place to protect the vitality of the French language in Quebec.
8. I think more legislation should be put in place to protect the vitality of the English language in Quebec.
9. I believe Indigenous languages of Canada should be considered official languages of Canada.
10. I believe there should be more legislation protecting the languages spoken by immigrants in Canada.
11. I think Quebec French is a good, valuable variety of French.
12. I think Quebec English is a good, valuable variety of English.
13. **English is a beautiful language.**
14. I feel motivated to speak English.
15. I like how English sounds.
16. **English is a language that is well-suited for modern society.**
17. English makes me feel secure.
18. **Knowing English will increase my opportunities to find employment.**
19. **English is more elegant than French.**
20. **Knowing English is a significant part of Canadian cultural heritage.**
21. **English is a language that is important to know in order to get far in life.**
22. **Knowing English is an important part of my personal identity.**
23. Speaking in English increases the value and prestige of what I say.
24. I prefer to speak English to comfort someone.
25. I like who I am when I speak English.
26. I feel true to myself when I speak English.
27. Speaking English gives me a sense of community.
28. When I am abroad, I look for English speakers to connect with.
29. I am likely to switch to English if a stranger interacting with me seems more comfortable in English.
30. In a new social context, I prefer to be addressed in English.
31. **French is a beautiful language.**
32. I feel motivated to speak French.
33. I like how French sounds.
34. **French is a language that is well-suited for modern society.**
35. French makes me feel secure.
36. **Knowing French will increase my opportunities to find employment.**
37. **French is more elegant than English.**
38. **Knowing French is a significant part of Canadian cultural heritage.**
39. **French is a language that is important to know in order to get far in life.**
40. **Knowing French is an important part of my personal identity.**
41. Speaking in French increases the value and prestige of what I say.
42. I prefer to speak French to comfort someone.
43. I like who I am when I speak French.
44. I feel true to myself when I speak French.
45. Speaking French gives me a sense of community.
46. When I am abroad, I look for French speakers to connect with.
47. I am likely to switch to French if a stranger interacting with me seems more comfortable in French.
48. In a new social context, I prefer to be addressed in French.

# B. Results of Principle Components Analysis

***Suitability for PCA***

Before implementing PCA, we assessed our dataset’s suitability for this analysis. First, we ran Barlett’s Test of Sphericity (Bartlett, 1954), which tests the overall significance of all correlations in the correlation matrix. Our test was significant (χ^2^ (1128) = 3902.28, *p* < 0.001), indicating that our data was appropriate for running PCA. Next, we calculated the Kaiser-Meyer-Olkin (KMO) index, which compares the magnitude of partial correlation coefficients relative to the total correlation coefficient (Cerny & Kaiser, 1977) and ranges from 0.50 to 1.00 (higher scores mean higher adequacy). The KMO index in our sample was 0.77, indicating suitability for PCA.

## Figure B1

*Parallel analysis scree plots revealing between four and five components*


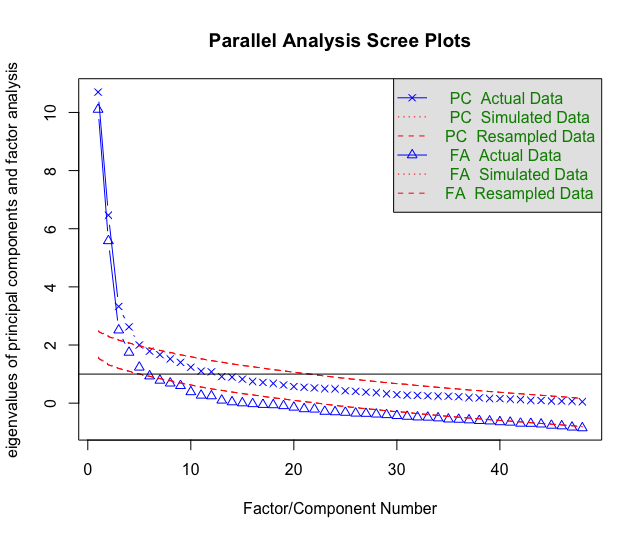


## Table B1

*Component Correlation Matrix*

| Component | 1 | 2 | 3 | 4 | 5 |
| --- | --- | --- | --- | --- | --- |
| 1 | 1.00 | -0.02 | 0.15 | 0.15 | 0.25 |
| 2 |  | 1.00 | 0.13 | 0.16 | -0.02 |
| 3 |  |  | 1.00 | 0.16 | -0.02 |
| 4 |  |  |  | 1.00 | 0.10 |
| 5 |  |  |  |  | 1.00 |

## Table B2

*Component Loadings for Language Attitude Items in LHQ (Five Components)*

| Item | Principal component | | | | |
| --- | --- | --- | --- | --- | --- |
|  | PC1 | PC2 | PC3 | PC4 | PC5 |
|  | French>  English Solidarity | English General | Minority Language Protection | French&  English Prestige | French Language Protection |
| French makes me feel secure. | **0.71** | 0.12 | 0.05 | -0.01 | 0.02 |
| I feel true to myself when I speak French. | **0.71** | 0.07 | -0.02 | 0.12 | 0.12 |
| I prefer to speak French to comfort someone. | **0.69** | -0.05 | -0.27 | -0.03 | 0.01 |
| In a new social context, I prefer to be addressed in French. | **0.65** | -0.20 | -0.11 | -0.01 | 0.13 |
| Knowing French is an important part of my personal identity.* | **0.60** | 0.14 | 0.23 | 0.09 | 0.16 |
| I feel motivated to speak French. | **0.59** | 0.15 | 0.23 | 0.03 | 0.14 |
| I like who I am when I speak French. | **0.59** | 0.15 | -0.06 | 0.11 | 0.16 |
| I like how French sounds. | **0.55** | 0.10 | 0.39 | 0.17 | 0.01 |
| Speaking French gives me a sense of community. | **0.53** | 0.13 | -0.05 | 0.17 | 0.27 |
| When I am abroad, I look for French speakers to connect with. | **0.52** | -0.12 | -0.16 | 0.14 | 0.18 |
| French is a beautiful language.* | **0.48** | 0.23 | 0.33 | 0.24 | 0.09 |
| I am likely to switch to French if a stranger interacting with me seems more comfortable in French. | **0.47** | 0.29 | 0.23 | 0.13 | -0.18 |
| I prefer to speak English to comfort someone. | **-0.65** | 0.19 | 0.12 | 0.29 | 0.06 |
| In a new social context, I prefer to be addressed in English. | **-0.54** | 0.24 | 0.11 | 0.30 | -0.14 |
| English is a beautiful language.* | 0.08 | **0.86** | -0.14 | -0.30 | 0.10 |
| I like how English sounds. | 0.06 | **0.85** | -0.07 | -0.19 | 0.00 |
| I feel motivated to speak English. | 0.06 | **0.81** | 0.13 | 0.02 | -0.07 |
| English is a language that is well-suited for modern society.* | 0.18 | **0.69** | -0.10 | 0.24 | -0.13 |
| English makes me feel secure. | -0.13 | **0.65** | 0.15 | 0.08 | -0.14 |
| Knowing English is an important part of my personal identity.* | -0.04 | **0.61** | 0.17 | 0.11 | 0.13 |
| I feel true to myself when I speak English. | **-0.43** | **0.50** | 0.14 | 0.18 | 0.09 |
| I am likely to switch to English if a stranger interacting with me seems more comfortable in English. | -0.01 | **0.50** | 0.16 | 0.25 | -0.12 |
| Knowing English is a significant part of Canadian cultural heritage.* | -0.13 | **0.48** | -0.03 | 0.22 | 0.18 |
| I like who I am when I speak English. | -0.24 | **0.43** | 0.01 | 0.32 | 0.20 |
| I am comfortable with English currently being the global language. | -0.18 | **0.42** | -0.12 | 0.39 | -0.12 |
| French is more elegant than English.* | 0.25 | -0.29 | 0.21 | **0.59** | 0.02 |
| Speaking in French increases the value and prestige of what I say. | 0.16 | -0.22 | -0.21 | **0.65** | 0.19 |
| I believe there should be more legislation protecting the Indigenous languages of Canada. | -0.09 | -0.11 | **0.74** | -0.14 | 0.26 |
| I believe there should be more legislation protecting the languages spoken by immigrants in Canada. | -0.10 | -0.15 | **0.66** | -0.07 | -0.15 |
| I believe Indigenous languages of Canada should be considered official languages of Canada. | -0.18 | -0.03 | **0.61** | -0.16 | 0.34 |
| I believe that people can have multiple identities at the same time. (E.g. Black + Canadian + Quebecer + English-speaking). | 0.04 | 0.19 | **0.61** | 0.04 | -0.03 |
| I value bilingualism. | 0.22 | 0.15 | **0.51** | 0.27 | -0.06 |
| English is more elegant than French.* | -0.35 | 0.39 | -0.31 | -0.37 | 0.07 |
| Speaking in English increases the value and prestige of what I say. | -0.29 | 0.01 | -0.27 | **0.54** | 0.19 |
| English is a language that is important to know in order to get far in life.* | 0.16 | 0.29 | 0.00 | **0.44** | -0.07 |
| Knowing French will increase my opportunities to find employment.* | 0.09 | 0.16 | 0.22 | 0.33 | 0.04 |
| I think Quebec French is a good, valuable variety of French. | 0.23 | 0.06 | 0.20 | -0.24 | **0.48** |
| I think more legislation should be put in place to protect the vitality of the French language in Quebec. | 0.09 | -0.09 | 0.02 | -0.08 | **0.65** |
| Knowing French is a significant part of Canadian cultural heritage.* | 0.03 | 0.18 | -0.02 | 0.23 | **0.61** |
| I agree with the laws protecting the French language in Quebec... | 0.19 | -0.02 | 0.21 | 0.08 | **0.58** |
| I support Bill 101. | 0.24 | -0.14 | 0.08 | 0.09 | **0.56** |
| French is a language that is important to know in order to get far in life.* | -0.13 | -0.02 | -0.14 | 0.32 | 0.36 |
| I think Quebec English is a good, valuable variety of English. | 0.27 | 0.11 | 0.11 | -0.04 | 0.26 |
| French is a language that is well-suited for modern society.* | 0.23 | 0.25 | 0.03 | 0.08 | 0.22 |
| Speaking English gives me a sense of community. | -0.28 | 0.36 | 0.03 | 0.20 | 0.13 |
| I think more legislation should be put in place to protect the vitality of the English language in Quebec. | -0.18 | 0.11 | 0.03 | 0.07 | -0.22 |
| When I am abroad, I look for English speakers to connect with. | -0.16 | 0.34 | 0.03 | -0.02 | -0.11 |
| Knowing English will increase my opportunities to find employment.* | 0.34 | 0.30 | 0.29 | 0.24 | -0.09 |
| Percentage of variance | 18.63 | 13.93 | 5.55 | 4.74 | 3.99 |
| Eigenvalue | 8.94 | 6.69 | 2.66 | 2.27 | 1.91 |

*Note.* Bold = component loadings with absolute values >= 0.40 after PCA with oblimin rotation and polychoric correlations.

* = Items in Kircher, 2009.

# C. Associations Between L1 and Latent Factors

We examined the associations between egos’ L1 and the four previously computed latent factors (*French Network, English Network, Bilingual Network, Ecology*) by fitting simple linear regressions with L1 as the independent variable (see Table C1). We found that L1 was a significant predictor of the *French Network* factor (*F*(2) = 13.42, *p* < 0.001) and the *English Network* factor (*F*(2) = 6.97, *p* = 0.01). Subsequent Tukey HSD post-hoc tests revealed that the L1 French group had a significantly larger, more interconnected, and strongly influential French-speaking personal network than the L1 English (HSD = 1.29, *p* < 0.001) or L1 French/English (HSD = -1.03, p < 0.001) groups. Conversely, the L1 French group also had a significantly smaller, less interconnected, and weakly influential English-speaking personal network than the other L1 groups (English vs. French HSD = -0.99, *p* = 0.01; French vs. French/English HSD = 0.68, *p* = 0.04).

## Table C1

*Mean Factor Loading Scores for Each L1 Group*

| L1 (*n* = 73) |  | Interpersonal and Ecological Language Dynamics | | | |
| --- | --- | --- | --- | --- | --- |
|  |  | French Network | English Network | Bilingual Network | Ecology |
| French  (*n* = 33) | Mean | **0.77** | **-0.53** | 0.04 | -0.23 |
|  | SE | 0.22 | 0.09 | 0.18 | 0.17 |
| English  (*n* = 19) | Mean | **-0.52** | **0.46** | -0.27 | 0.38 |
|  | SE | 0.01 | 0.26 | 0.21 | 0.19 |
| French/English  (*n* = 21) | Mean | **-0.26** | **0.15** | 0.25 | -0.02 |
|  | SE | 0.17 | 0.29 | 0.23 | 0.23 |
|  | *Df* | 2 | 2 | 2 | 2 |
|  | *F-value* | 13.42 | 6.97 | 1.28 | 2.47 |
|  | *p-value* | **<0.001** | **0.01** | 0.28 | 0.09 |

*Note.* Bold = significant at *p* < 0.05.

# D. Predicting Language Attitudes Based on Predefined Categories

In our manuscript, we took a bottom-up, data-driven approach using PCA to assess patterns underlying our language attitudes questionnaire. Here, we broke down the language attitudes items using a top-down approach based on the classification by Kircher (2009), which is grounded in the dimensions proposed by previous literature (i.e., general, status, and solidarity). Specifically, we regrouped the items in two ways: one only using the items in Kircher (2009), and the other using the same items plus additional items in our questionnaire relating to English and French (Table D1). After dividing the items into these predefined categories, we computed the mean for each dimension of language attitudes (e.g., French Status), which were the dependent variables in our following robust multiple linear regression models (Table D2-D4).

## Table D1

*Categorization of Language Attitude Items*

| Categories | French | English |
| --- | --- | --- |
| General | **French is a beautiful language.** | **English is a beautiful language.** |
|  | I feel motivated to speak French. | I feel motivated to speak English. |
|  | I like how French sounds. | I like how English sounds. |
|  | **French is more elegant than English.** | **English is more elegant than French.** |
| Status | **French is a language that is well-suited for modern society.** | **English is a language that is well-suited for modern society.** |
|  | **Knowing French will increase my opportunities to find employment.** | **Knowing English will increase my opportunities to find employment.** |
|  | **French is a language that is important to know in order to get far in life.** | **English is a language that is important to know in order to get far in life.** |
|  | Speaking in French increases the value and prestige of what I say. | Speaking in English increases the value and prestige of what I say. |
| Solidarity | I think more legislation should be put in place to protect the vitality of the French language in Quebec. | I think more legislation should be put in place to protect the vitality of the English language in Quebec. |
|  | I think Quebec French is a good, valuable variety of French. | I think Quebec English is a good, valuable variety of English. |
|  | French makes me feel secure. | English makes me feel secure. |
|  | **Knowing French is a significant part of Canadian cultural heritage.** | **Knowing English is a significant part of Canadian cultural heritage.** |
|  | **Knowing French is an important part of my personal identity.** | **Knowing English is an important part of my personal identity.** |
|  | I prefer to speak French to comfort someone. | I prefer to speak English to comfort someone. |
|  | I like who I am when I speak French. | I like who I am when I speak English. |
|  | I feel true to myself when I speak French. | I feel true to myself when I speak English. |
|  | Speaking French gives me a sense of community. | Speaking English gives me a sense of community. |
|  | When I am abroad, I look for French speakers to connect with. | When I am abroad, I look for English speakers to connect with. |
|  | I am likely to switch to French if a stranger interacting with me seems more comfortable in French. | I am likely to switch to English if a stranger interacting with me seems more comfortable in English. |
|  | In a new social context, I prefer to be addressed in French. | In a new social context, I prefer to be addressed in English. |
| Minority Languages | I believe that people can have multiple identities at the same time. (E.g. Black + Canadian + Quebecer + English-speaking). | |
|  | I believe there should be more legislation protecting the Indigenous languages of Canada. | |
|  | I believe Indigenous languages of Canada should be considered official languages of Canada. | |
|  | I believe there should be more legislation protecting the languages spoken by immigrants in Canada. | |
|  | I value bilingualism. | |

*Note.* Bold = items in Kircher (2009).

## Table D2

*Robust Multiple Linear Regressions Predicting Language Attitudes Broken Down by General, Status, and Solidarity (Kircher)*

|  | French General | | | | | | French Status | | | | | | French Solidarity | | | | | |
| --- | --- | --- | --- | --- | --- | --- | --- | --- | --- | --- | --- | --- | --- | --- | --- | --- | --- | --- |
| Predictor | *B* | *SE B* | *β* | 95% CI | *t* | *p* | *B* | *SE B* | *β* | 95% CI | *t* | *p* | *B* | *SE B* | *β* | 95% CI | *t* | *p* |
| French Factor | 0.30 | 0.11 | 0.34 | [ 0.07, 0.52] | 2.63 | **0.01** | 0.11 | 0.08 | 0.17 | [-0.04, 0.27] | 1.44 | 0.15 | 0.21 | 0.10 | 0.25 | [ 0.01, 0.41] | 2.08 | **0.04** |
| English Factor | -0.04 | 0.12 | -0.04 | [-0.27, 0.19] | -0.34 | 0.74 | -0.01 | 0.08 | -0.01 | [-0.17, 0.15] | -0.08 | 0.94 | -0.11 | 0.10 | -0.12 | [-0.31, 0.10] | -1.02 | 0.31 |
| Bilingual Factor | 0.18 | 0.12 | 0.19 | [-0.05, 0.41] | 1.54 | 0.13 | 0.12 | 0.08 | 0.17 | [-0.04, 0.29] | 1.54 | 0.13 | 0.03 | 0.10 | 0.03 | [-0.18, 0.23] | 0.26 | 0.79 |
| Ecology | 0.39 | 0.12 | 0.39 | [ 0.14, 0.63] | 3.16 | **0.00** | 0.24 | 0.09 | 0.31 | [ 0.07, 0.41] | 2.80 | **0.01** | 0.08 | 0.11 | 0.08 | [-0.14, 0.29] | 0.69 | 0.49 |
| *R²* |  |  |  | 0.20 |  |  |  |  |  | 0.13 |  |  |  |  |  | 0.11 |  |  |

|  | English General | | | | | | English Status | | | | | | English Solidarity | | | | | |
| --- | --- | --- | --- | --- | --- | --- | --- | --- | --- | --- | --- | --- | --- | --- | --- | --- | --- | --- |
| Predictor | *B* | *SE B* | *β* | 95% CI | *t* | *p* | *B* | *SE B* | *β* | 95% CI | *t* | *p* | *B* | *SE B* | *β* | 95% CI | *t* | *p* |
| French Factor | -0.28 | 0.14 | -0.29 | [-0.56, 0.00] | -2.02 | **0.047** | 0.13 | 0.09 | 0.19 | [-0.06, 0.31] | 1.36 | 0.18 | -0.06 | 0.12 | -0.06 | [-0.29, 0.18] | -0.49 | 0.63 |
| English Factor | -0.06 | 0.14 | -0.06 | [-0.34, 0.23] | -0.41 | 0.69 | -0.10 | 0.09 | -0.14 | [-0.28, 0.09] | -1.00 | 0.32 | 0.08 | 0.12 | 0.08 | [-0.16, 0.32] | 0.63 | 0.53 |
| Bilingual Factor | -0.01 | 0.14 | -0.01 | [-0.30, 0.27] | -0.10 | 0.92 | 0.08 | 0.10 | 0.12 | [-0.11, 0.27] | 0.89 | 0.38 | -0.04 | 0.12 | -0.04 | [-0.28, 0.20] | -0.32 | 0.75 |
| Ecology | -0.19 | 0.15 | -0.17 | [-0.49, 0.11] | -1.26 | 0.21 | 0.12 | 0.10 | 0.16 | [-0.08, 0.32] | 1.21 | 0.23 | 0.02 | 0.13 | 0.02 | [-0.24, 0.27] | 0.14 | 0.89 |
| *R²* |  |  |  | 0.10 |  |  |  |  |  | 0.09 |  |  |  |  |  | 0.02 |  |  |

*Note.* Bold = significant at *p* < 0.05.

## Table D3

*Robust Multiple Linear Regressions Predicting Language Attitudes Broken Down by General, Status, and Solidarity (Kircher and Additional Items)*

|  | French General | | | | | | French Status | | | | | | French Solidarity | | | | | |
| --- | --- | --- | --- | --- | --- | --- | --- | --- | --- | --- | --- | --- | --- | --- | --- | --- | --- | --- |
| Predictor | *B* | *SE B* | *β* | 95% CI | *t* | *p* | *B* | *SE B* | *β* | 95% CI | *t* | *p* | *B* | *SE B* | *β* | 95% CI | *t* | *p* |
| French Factor | 0.26 | 0.10 | 0.32 | [ 0.06, 0.46] | 2.60 | **0.01** | 0.18 | 0.09 | 0.26 | [ 0.00, 0.35] | 2.03 | **0.047** | 0.31 | 0.11 | 0.34 | [ 0.09, 0.52] | 2.87 | **0.01** |
| English Factor | -0.05 | 0.10 | -0.06 | [-0.26, 0.15] | -0.52 | 0.61 | -0.06 | 0.09 | -0.09 | [-0.24, 0.12] | -0.70 | 0.49 | -0.15 | 0.11 | -0.16 | [-0.37, 0.07] | -1.38 | 0.17 |
| Bilingual Factor | 0.14 | 0.10 | 0.16 | [-0.07, 0.34] | 1.33 | 0.19 | 0.16 | 0.09 | 0.22 | [-0.02, 0.34] | 1.81 | 0.08 | 0.18 | 0.11 | 0.19 | [-0.04, 0.40] | 1.67 | 0.10 |
| Ecology | 0.22 | 0.11 | 0.24 | [ 0.00, 0.43] | 2.01 | **0.048** | 0.24 | 0.09 | 0.31 | [ 0.05, 0.43] | 2.58 | **0.01** | -0.01 | 0.12 | -0.01 | [-0.24, 0.22] | -0.09 | 0.93 |
| *R²* |  |  |  | 0.15 |  |  |  |  |  | 0.18 |  |  |  |  |  | 0.19 |  |  |

|  | English General | | | | | | English Status | | | | | | English Solidarity | | | | | |
| --- | --- | --- | --- | --- | --- | --- | --- | --- | --- | --- | --- | --- | --- | --- | --- | --- | --- | --- |
| Predictor | *B* | *SE B* | *β* | 95% CI | *t* | *p* | *B* | *SE B* | *β* | 95% CI | *t* | *p* | *B* | *SE B* | *β* | 95% CI | *t* | *p* |
| French Factor | -0.22 | 0.12 | -0.24 | [-0.46, 0.03] | -1.76 | 0.08 | 0.08 | 0.08 | 0.12 | [-0.08, 0.24] | 1.01 | 0.32 | -0.17 | 0.08 | -0.23 | [-0.33, 0.00] | -2.02 | **0.048** |
| English Factor | -0.09 | 0.13 | -0.10 | [-0.34, 0.16] | -0.72 | 0.47 | -0.12 | 0.08 | -0.18 | [-0.29, 0.04] | -1.50 | 0.14 | 0.07 | 0.08 | 0.09 | [-0.10, 0.23] | 0.79 | 0.43 |
| Bilingual Factor | -0.02 | 0.13 | -0.02 | [-0.27, 0.23] | -0.14 | 0.89 | 0.14 | 0.08 | 0.19 | [-0.03, 0.30] | 1.67 | 0.10 | -0.03 | 0.08 | -0.03 | [-0.19, 0.14] | -0.30 | 0.76 |
| Ecology | -0.08 | 0.13 | -0.08 | [-0.35, 0.18] | -0.64 | 0.52 | 0.09 | 0.09 | 0.13 | [-0.08, 0.27] | 1.09 | 0.28 | 0.16 | 0.09 | 0.19 | [-0.02, 0.33] | 1.75 | 0.09 |
| *R²* |  |  |  | 0.04 |  |  |  |  |  | 0.09 |  |  |  |  |  | 0.13 |  |  |

*Note.* Bold = significant at *p* < 0.05.

## Table D4

*Robust Multiple Linear Regressions Predicting Language Attitudes Towards Minority Languages*

| Predictor | *B* | *SE B* | *β* | 95% CI | *t* | *p* |
| --- | --- | --- | --- | --- | --- | --- |
| French Factor | -0.01 | 0.09 | -0.01 | [-0.18, 0.16] | -0.12 | 0.91 |
| English Factor | -0.01 | 0.09 | -0.02 | [-0.19, 0.16] | -0.14 | 0.89 |
| Bilingual Factor | -0.12 | 0.09 | -0.16 | [-0.29, 0.06] | -1.35 | 0.18 |
| Ecology | 0.14 | 0.09 | 0.19 | [-0.04, 0.33] | 1.56 | 0.12 |
| *R²* |  |  |  | 0.07 |  |  |

# E. Results for Four Components

## Table E1

*Component Loadings for Language Attitude Items in LHQ (Four Components)*

| Item | Principal component | | | |
| --- | --- | --- | --- | --- |
|  | PC1 | PC2 | PC3 | PC4 |
|  | French>  English Solidarity | English General | Minority Language Protection | French&  English Prestige |
| I feel true to myself when I speak French. | **0.75** | 0.07 | -0.02 | 0.10 |
| French makes me feel secure. | **0.72** | 0.14 | 0.02 | -0.06 |
| I prefer to speak French to comfort someone. | **0.70** | -0.05 | -0.28 | -0.06 |
| In a new social context, I prefer to be addressed in French. | **0.70** | -0.22 | -0.10 | 0.00 |
| Knowing French is an important part of my personal identity... | **0.65** | 0.15 | 0.24 | 0.08 |
| I like who I am when I speak French. | **0.65** | 0.14 | -0.05 | 0.12 |
| I feel motivated to speak French. | **0.64** | 0.15 | 0.23 | 0.01 |
| Speaking French gives me a sense of community. | **0.62** | 0.11 | -0.01 | 0.21 |
| When I am abroad, I look for French speakers to connect with. | **0.58** | -0.13 | -0.14 | 0.17 |
| I like how French sounds. | **0.56** | 0.16 | 0.35 | 0.09 |
| French is a beautiful language. | **0.52** | 0.27 | 0.31 | 0.19 |
| I support Bill 101. | **0.43** | -0.23 | 0.21 | 0.26 |
| I am likely to switch to French if a stranger interacting with me seems more comfortable in French. | **0.41** | 0.37 | 0.14 | 0.00 |
| I prefer to speak English to comfort someone. | **-0.63** | 0.20 | 0.14 | 0.32 |
| In a new social context, I prefer to be addressed in English. | **-0.58** | 0.29 | 0.07 | 0.26 |
| I feel motivated to speak English. | 0.04 | **0.84** | 0.08 | -0.03 |
| I like how English sounds. | 0.07 | **0.83** | -0.09 | -0.20 |
| English is a beautiful language. | 0.11 | **0.80** | -0.13 | -0.26 |
| English is a language that is well-suited for modern society. | 0.15 | **0.73** | -0.16 | 0.16 |
| English makes me feel secure. | -0.17 | **0.69** | 0.10 | 0.01 |
| Knowing English is an important part of my personal identity. | 0.00 | **0.60** | 0.18 | 0.13 |
| I am likely to switch to English if a stranger interacting with me seems more comfortable in English. | -0.04 | **0.55** | 0.10 | 0.17 |
| I feel true to myself when I speak English. | -0.39 | **0.49** | 0.16 | 0.21 |
| I am comfortable with English currently being the global language. | -0.21 | **0.47** | -0.17 | 0.32 |
| Knowing English is a significant part of Canadian cultural heritage. | -0.06 | **0.46** | 0.00 | 0.27 |
| I like who I am when I speak English. | -0.16 | **0.41** | 0.05 | 0.37 |
| I believe there should be more legislation protecting the Indigenous languages of Canada. | -0.01 | -0.13 | **0.80** | -0.08 |
| I believe Indigenous languages of Canada should be considered official languages of Canada. | -0.08 | -0.08 | **0.70** | -0.06 |
| I believe there should be more legislation protecting the languages spoken by immigrants in Canada. | -0.15 | -0.09 | **0.62** | -0.15 |
| I believe that people can have multiple identities at the same time. (E.g. Black + Canadian + Quebecer + English-speaking). | 0.03 | 0.24 | **0.58** | -0.02 |
| I value bilingualism. | 0.20 | 0.23 | **0.46** | 0.17 |
| Speaking in French increases the value and prestige of what I say. | 0.23 | -0.20 | -0.18 | **0.67** |
| Speaking in English increases the value and prestige of what I say. | -0.22 | 0.00 | -0.23 | **0.61** |
| French is more elegant than English. | 0.27 | -0.21 | 0.19 | **0.52** |
| French is a language that is important to know in order to get far in life. | -0.01 | -0.07 | -0.05 | **0.44** |
| Knowing French is a significant part of Canadian cultural heritage. | 0.23 | 0.08 | 0.12 | **0.42** |
| English is a language that is important to know in order to get far in life. | 0.15 | 0.35 | -0.04 | 0.36 |
| Knowing French will increase my opportunities to find employment. | 0.11 | 0.20 | 0.21 | 0.29 |
| I agree with the laws protecting the French language in Quebec. | 0.38 | -0.11 | 0.34 | 0.24 |
| Speaking English gives me a sense of community. | -0.23 | 0.35 | 0.06 | 0.24 |
| I think more legislation should be put in place to protect the vitality of the French language in Quebec. | 0.30 | -0.22 | 0.19 | 0.15 |
| Knowing English will increase my opportunities to find employment. | 0.32 | 0.37 | 0.23 | 0.13 |
| French is a language that is well-suited for modern society. | 0.30 | 0.22 | 0.06 | 0.13 |
| I think Quebec English is a good, valuable variety of English. | 0.35 | 0.06 | 0.16 | 0.02 |
| I think more legislation should be put in place to protect the vitality of the English language in Quebec. | -0.25 | 0.16 | -0.02 | 0.00 |
| When I am abroad, I look for English speakers to connect with. | -0.19 | 0.35 | 0.00 | -0.06 |
| I think Quebec French is a good, valuable variety of French. | 0.38 | -0.04 | 0.32 | -0.09 |
| English is more elegant than French. | -0.33 | 0.30 | -0.27 | -0.28 |
| Percentage of variance | 18.62 | 13.93 | 5.55 | 4.74 |
| Eigenvalue | 8.94 | 6.68 | 2.66 | 2.27 |

*Note.* Bold = component loadings with absolute values >= 0.40 after PCA with oblimin rotation and polychoric correlations.

## Table E2

*Mean Component Scores for L1 Groups*

| L1(*N =* 123) |  | Principal component | | | |
| --- | --- | --- | --- | --- | --- |
|  |  | French>  English Solidarity (PC1) | English General (PC2) | Minority Language Protection (PC3) | French>  English  Prestige (PC4) |
| French  (*n* = 56) | Mean | 0.50 | 0.00 | -0.06 | -0.03 |
|  | SE | 0.13 | 0.14 | 0.14 | 0.16 |
| English  (*n* = 32) | Mean | -0.95 | -0.14 | -0.10 | 0.06 |
|  | SE | 0.19 | 0.20 | 0.17 | 0.16 |
| French/English  (*n* = 35) | Mean | -0.06 | 0.13 | 0.19 | -0.01 |
|  | SE | 0.14 | 0.15 | 0.14 | 0.18 |
|  | *Df* | 2 | 2 | 2 | 2 |
|  | *F-value* | 22.48 | 0.58 | 1.01 | 0.08 |
|  | *p-value* | **<0.001** | 0.56 | 0.37 | 0.93 |

*Note.* Bold = significant at *p* < 0.05.

## Table E3

*Robust Multiple Linear Regressions Predicting Language Attitudes Component Scores*

|  | French>English Solidarity | | | | | | English General | | | | | |
| --- | --- | --- | --- | --- | --- | --- | --- | --- | --- | --- | --- | --- |
| Predictor | *B* | *SE B* | *β* | 95% CI | *t* | *p* | *B* | *SE B* | *β* | 95% CI | *t* | *p* |
| French Factor | 0.43 | 0.13 | 0.39 | [ 0.17, 0.70] | 3.30 | **0.002** | -0.16 | 0.12 | -0.18 | [-0.41, 0.08] | -1.33 | 0.19 |
| English Factor | -0.17 | 0.14 | -0.15 | [-0.44, 0.10] | -1.28 | 0.20 | -0.01 | 0.13 | -0.01 | [-0.26, 0.24] | -0.10 | 0.92 |
| Bilingual Factor | 0.23 | 0.14 | 0.20 | [-0.04, 0.50] | 1.72 | 0.09 | 0.00 | 0.13 | 0.00 | [-0.25, 0.25] | 0.02 | 0.99 |
| Ecology | -0.02 | 0.14 | -0.01 | [-0.30, 0.27] | -0.11 | 0.91 | 0.03 | 0.13 | 0.03 | [-0.23, 0.30] | 0.24 | 0.81 |
| *R²* |  |  |  | 0.24 |  |  |  |  |  | 0.03 |  |  |

|  | Minority Language Protection | | | | | | French&English Prestige | | | | | |
| --- | --- | --- | --- | --- | --- | --- | --- | --- | --- | --- | --- | --- |
| Predictor | *B* | *SE B* | *β* | *95% CI* | *t* | *p* | *B* | *SE B* | *β* | *95% CI* | *t* | *p* |
| French Factor | -0.06 | 0.10 | -0.08 | [-0.27, 0.14] | -0.63 | 0.53 | 0.09 | 0.10 | 0.10 | [-0.12, 0.30] | 0.84 | 0.41 |
| English Factor | 0.01 | 0.10 | 0.02 | [-0.19, 0.22] | 0.12 | 0.91 | -0.03 | 0.11 | -0.02 | [-0.24, 0.19] | -0.26 | 0.80 |
| Bilingual Factor | -0.21 | 0.10 | -0.24 | [-0.42, 0.00] | -1.98 | 0.05 | 0.09 | 0.11 | 0.09 | [-0.12, 0.31] | 0.86 | 0.39 |
| Ecology | 0.24 | 0.11 | 0.27 | [ 0.02, 0.46] | 2.20 | **0.03** | 0.37 | 0.11 | 0.35 | [ 0.14, 0.59] | 3.23 | **0.002** |
| *R²* |  |  |  | 0.14 |  |  |  |  |  | 0.08 |  |  |

*Note.* Bold = significant at *p* < 0.05.

# F. References

Bartlett, M. S. (1954). A Note on the Multiplying Factors for Various χ2 Approximations. *Journal of the Royal Statistical Society: Series B (Methodological)*, *16*(2), 296–298. https://doi.org/10.1111/j.2517-6161.1954.tb00174.x

Cerny, B. A., & Kaiser, H. F. (1977). A Study Of A Measure Of Sampling Adequacy For Factor-Analytic Correlation Matrices. *Multivariate Behavioral Research*, *12*(1), 43–47. https://doi.org/10.1207/s15327906mbr1201_3

Kircher, R. (2009). *Language attitudes in Quebec: A contemporary perspective* [Thesis, Queen Mary University of London]. https://qmro.qmul.ac.uk/xmlui/handle/123456789/497
